# *DRD2* and *DRD4* genes related to cognitive deficits in HIV-infected adults who abuse alcohol

**DOI:** 10.1186/s12993-015-0072-x

**Published:** 2015-08-27

**Authors:** Karina Villalba, Jessy G. Devieux, Rhonda Rosenberg, Jean Lud Cadet

**Affiliations:** Department of Health Promotion and Disease Prevention, Robert Stempel College of Public Health and Social Work, Florida International University, Biscayne Bay Campus, 3000 N.E, 151 Street ACI #260, North Miami, FL 33181 USA; NIDA Intramural Program, Molecular Neuropsychiatry Research Branch, Baltimore, MD USA

**Keywords:** Dopamine, *DRD2* gene, *DRD4* gene, HIV, Alcohol abuse

## Abstract

**Background:**

HIV-infected individuals continue to experience neurocognitive deterioration despite virologically successful treatments. The causes of neurocognitive impairment are still unclear. However, several factors have been suggested including the role of genetics. There is evidence suggesting that neurocognitive impairment is heritable and individual differences in cognition are strongly driven by genetic variations. The contribution of genetic variants affecting the metabolism and activity of dopamine may influence these individual differences.

**Methods:**

The present study explored the relationship between two candidate genes *(DRD4* and *DRD2*) and neurocognitive performance in HIV-infected adults. A total of 267 HIV-infected adults were genotyped for polymorphisms, *DRD4* 48 bp-variable number tandem repeat (VNTR), *DRD2* rs6277 and *ANKK1* rs1800497. The Short Category (SCT), Color Trail (CTT) and Rey-Osterrieth Complex Figure Tests (ROCT) were used to measure executive function and memory.

**Results:**

Results showed significant associations with the SNP rs6277 and impaired executive function (odds ratio = 3.3, 95 % CI 1.2–2.6; p = 0.004) and cognitive flexibility (odds ratio = 1.6, 95 % CI 2.0–5.7; p = 0.001). The results were further stratified by race and sex and significant results were seen in males (odds ratio = 3.5, 95 % CI 1.5–5.5; p = 0.008) and in African Americans (odds ratio = 3.1, 95 % CI 2.3–3.5; p = 0.01). Also, *DRD4* VNTR 7-allele was significantly associated with executive dysfunction.

**Conclusion:**

The study shows that genetically determined differences in the SNP rs6277 *DRD2* gene and *DRD4* 48 bp VNTR may be risk factors for deficits in executive function and cognitive flexibility.

## Background

Human immunodeficiency virus (HIV) is a global epidemic that affects approximately 36 million people worldwide [1]. In addition to its deleterious effects on the cell-mediated immune system, HIV can also damage cells in the central nervous system and lead to HIV-associated neurocognitive disorders (HAND) [2]. The manifestations of HAND have significantly changed in response to the introduction of antiretroviral therapy (ART). For example, the incidence of HIV-associated dementia has declined. However, the prevalence of asymptomatic and mild neurocognitive impairment have increased with increased longevity [3]. HAND encompasses a wide range of cognitive impairment that includes deficient memory and attention, decreased executive function, and behavioral changes, such as apathy or lethargy [4].

Cognitive control processes regulating thought and action are multifaceted functions influenced by heritable genetic factors and environmental influences [5]. Individuals increasingly select and modify their experiences partly based on their genetic predispositions [6–8]. Friedman et al. indicated that individual differences in executive function including inhibiting dominant responses, updating working memory representations, and shifting between task sets, are almost 99 % heritable [7]. Cognitive neuroscience and pharmacology associate dopamine and serotonin as neuromodulators of cognition [5]. Furthermore, studies found associations between dopamine polymorphisms with sustained attention, memory, and executive function phenotypes in both clinical and non-clinical populations [9–12].

Dopamine neurons are located in the ventral midbrain and are involved in several cognitive functions that influence performance, motor control, reward, and cognition [13–15]. Dopamine modulates executive function by co-jointly adjusting neurochemical transmission in the prefrontal cortex (PFC) [5, 16]. The PFC plays a central role in the top-down control of many higher-order executive tasks. It is involved in learning, memory, categorization, inhibition control, and cognitive flexibility [17, 18]. Activation of D1, D2, D3, and D4 receptors modulate the excitability of receptor cells and PFC neural network activity. [19] The SNP rs1800497 (also known as TaqIA) of the D2 receptor gene DRD2 is one of the most extensively investigated genes related to neuropsychiatric disorders [19, 20]. This DRD2-associated polymorphism is located within the coding region of a neighboring gene, *ANKK1* and is associated with a reduced number of dopamine binding sites in the brain [21]. The SNP rs1800497 is located more than ten kilobase-pairs downstream from the coding region of the *DRD2* gene in chromosome 11q23 and is, therefore, unlikely to alter *DRD2* directly [22]. Proximity of the two genes may reflect functional relationship and may be associated with dopaminergic phenotypes by being in linkage disequilibrium [6, 23]. Polymorphism *DRD2* SNP rs6277 has been reported to affect D2 receptor density in the striatum [24]. Several studies have shown that SNP rs6277 is associated with prefrontal cortex-mediated behaviors including attentional control, planning and verbal reasoning [20]. A study on cognitive flexibility showed that SNP rs6277 was a strong predictor of learning from negative reward prediction errors by avoiding those responses linked to negative outcomes [6, 25].

The dopamine D4 receptor is widely expressed in the central nervous system, particularly in the frontal cortex, hippocampus, amygdala and hypothalamus [15, 26]. The dopamine D4 receptor *DRD4* gene is located on chromosome 11p15.5 and has a highly variable number of tandem repeats in the coding sequence [27]. The polymorphism is a 48 bp VNTR sequence in exon 3, encoding the third intracellular loop of D4 receptor [28]. The most common polymorphic variants of the receptor are D4.7, and D4.4 [29, 30]. Individuals with D4.7 repeat show both reduced binding affinities and receptor densities for dopamine neurotransmission [31]. The D4.7 repeat is correlated with impulsivity and lower levels of response inhibition [32]. Several studies have analyzed the association between the D4.7-repeat allele in *DRD4* gene and attention-deficit hyperactivity disorder (ADHD) [10, 26].

Memory deficits and executive dysfunction are highly prevalent among HIV-infected adults [33]. These conditions can affect their quality of life, antiretroviral adherence, and HIV risk behaviors [34]. The causes of asymptomatic neurocognitive impairment are still unclear. However, several factors have been suggested including the role of genetics [33]. Cognitive functions are influenced by dopamine. Thus, genetic differences in the dopamine system genes may exacerbate the development of neurocognitive impairment in an individual [5, 35]. The present study explored potential associations with *DRD2* rs6277, *ANNK1* rs1800497 and *DRD4* 48 bp VNTR polymorphism and cognitive functions in HIV-infected adults.

## Methods

### Participants

This study utilized a cross-sectional design, using baseline data gathered between 2009 and 2012 as part of a longitudinal randomized controlled trial for reducing risk behaviors among HIV-infected alcohol abusers. The main study recruited a total of 379 individuals. However, the current study used 267 biologically-unrelated individuals, because 112 participants declined to provide blood samples for genetic testing. Recruitment was made in a multicultural, low income, urban areas of Miami-Dade County, Florida. Participants were between 18 and 60 years of age, HIV-positive and willing to present documentation to confirm serostatus, consumed alcohol within the last 3 months, with a history of alcohol abuse or dependence within the past 2 years, and, at the time of recruitment, were not showing overt signs of major psychiatric disorders. Additionally, availability to provide a blood specimen was required. All participants provided signed informed consent as approved by the Institutional Review Board (IRB) at Florida International University.

Participants were evaluated for alcohol use by the Timeline Followback (TLFB) and the Alcohol Use Disorders Identification Test (AUDIT) test. All participants were assessed using the same battery of neurocognitive tests and in the same order. Nonverbal memory was measured with the Rey-Osterrieth Complex Figure Tests (RCFT). Cognitive flexibility was measured with the Color Trails (CTT B), and executive function was measured by the Short Category Test (SCT).

### Genotyping

DNA was extracted from whole blood by manual extraction using the QIAamp DNA Mini Kit (Valencia, CA). SNPs rs6277 and rs1800497 were genotyped using the TaqMan^®^ SNP Genotyping Assays (Foster City, CA, USA). Allelic discrimination analysis was performed on the Bio-Rad CFX96™ real-time PCR machine (Hercules, CA, USA).

For VNTR D4, Bio-Rad CFX Manager software (version 3.0) was used for data acquisition and genotype assignment. The primer sequences used for the D4 amplification were obtained from a previous study [36]. The sequence was as follows: 5′ CTGCTGCTCTACTGGGC 3′ sense and 5′ GTGCACCACGAAGGAAGG 3′ antisense The 25 μl reaction mixture contained: 1× PCR amplification buffer (Qiagen, Valencia, CA, USA), 300 μM dNTPs, 0.5 μM of each primer, 0.5 U Taq DNA polymerase (Qiagen) and 50 ng of genomic DNA. The temperature cycle consisted of an initial denaturation at 94 °C for 5 min, followed by 30 cycles of annealing for 40 s at 54 °C, extension for 40 s at 72 °C, denaturing for 40 s at 94 °C, and then the final extension for 6 min at 72 °C. The amplification products were separated on a 3 % agarose gel electrophoresis according to the number of repeats. The size of the amplified fragments was from 500 to 750 bp (2–7 copies of the 48pb repeat). These genetic markers were chosen based on prior evidence of the SNPs conferring risk to neurocognitive deficits or a theoretical association with executive function.

### Neurocognitive measures

The neurocognitive test battery included standardized measures of multiple domains of cognitive function selected for their sensitivity to HIV-associated neurocognitive impairment. The neurocognitive tests were assessed in the following domains:

1. *VisualMemory*-Rey ROCT evaluated visuospatial construction and nonverbal memory [37]. It consists of a complex geometric figure that is copied and then redrawn from memory [38]. Copy and accuracy of correctly copied or recalled elements were measured based on a score from 0 to 36. The figure was divided into 18 components. Each piece was evaluated with respect to its drawing accuracy with higher scores indicating better accuracy.
2. *Cognitive flexibility* CTT-B evaluated cognitive flexibility. Participants were presented with numbered colored circles that required starting with a pink colored number one circle and alternating between pink and yellow colored circles as fast as possible [39]. The test measured time in seconds to complete, with higher scores indicating poor performance. High test–retest reliability scores ranging from 0.85 to 1.00 [39].
3. *Executive function* SCT evaluated executive function. It consisted of five booklets with 20 cards per subtest and required the individual to formulate an organizing concept for each subtest. The number of errors on each booklet was added and the total number of errors determined impairment with lower scores representing better executive function [40]. Test–retest coefficients range from 0.60 to 0.96 depending upon the severity of impairment in the sample.

Neurocognitive tests were completed at baseline. Trained personnel administered the tests in the same order and according to standardized procedures.

### Alcohol use

The TLFB method assessed alcohol use and other drugs of abuse. This method obtains estimates of substance use by using a calendar format and providing retrospective estimates of the participant’s substance use over the last 3 months [41]. The AUDIT is a screening tool that is sensitive to early detection of high-risk drinking behaviors [42].

## Analysis

Since 112 (29 %) individuals did not to participate in this study, thus, data were evaluated for potential selection bias. Statistical analyses were performed using Stata v.11 (StataCorp, College Station, TX, USA). Logistic and linear regression methods were used to calculate crude and multifactorial (self- reported ethnicity/race, alcohol use severity, viral load, CD4 count, cannabis and cocaine use) adjusted odds ratios (OR), including a 95 % confidence intervals (CIs) and test for interaction. All statistical tests were two-tailed, and the threshold for statistical significance was set at *P* < 0.05. Ethnic and gender -specific associations were calculated through stratified analyses. Genotyping counts were tested for Hardy–Weinberg equilibrium using an exact test. For the *DRD4* polymorphism, the Pearson’s X^2^ and Student’s t test were used to compare group differences. For *DRD4* 48 bp VNTR, alleles were grouped in short (S; <7 repeat) and long (L; ≥7 repeat) as described in previous studies [43, 44]. For statistical analysis, participants were placed in one of two genotype groups 7-allele present (homozygous for the short allele) or 7-allele absent (heterozygous or homozygous for the long allele).

To standardize cognitive measures for this study, standardized T-*scores* were developed by using multiple linear regression methods analyzing the influence of age, sex, education, and ethnicity on each cognitive test score. Each of the cognitive domains was included as dependent variables. The continuous predictor was age, and the categorical predictors were sex, education and race/ethnicity. For each regression, all the predictors were included in the model, retaining only the variables that significantly contributed to the prediction of cognitive test score. The β weights of each of these predictors in the final model, as well as the standard error of each regression model, were used to calculate predicted scores on each test. These predictive scores were subtracted from each individual actual composite score to calculate residual scores. Residual scores were then converted to *T*-*scores* (mean 50; SD = 10). T-*scores* were used to determine cognitive impairment according to the Frascati criteria [45], as shown in Table 1. For the cognitive domains, scores were developed as follows: executive function (SCT), visual memory (RCFT) and cognitive flexibility (CTT-B).

**Table 1:** Categories of HIV-associated neurocognitive disorder according to Frascati criteria

	Neurocognitive status^a^	Functional status^b^
Asymptomatic neurocognitive impairment	1 SD below the mean in 2 cognitive domains	No impairment in activities of daily living
Mild neurocognitive impairment or disorder	1 SD below the mean in 2 cognitive domains	Impairment in activities of daily living
HIV-associated dementia	2 SD below the mean in 2 cognitive domains	Notable impairment in activities of daily living

*SD* standard deviation

^a^Neurocognitive testing should include an assessment of at least five domains, including attention–information processing, language, abstraction-executive, complex perceptual motor skills, memory (including learning and recall), simple motor skills, or sensory, perceptual skills

^b^No agreed measures exist for HIV-associated neurocognitive disorder criteria

## Results

Of the 379 participants recruited for the main study, 70 % (N = 267) provided blood samples. The participants were 94 (34 %) females and 173 (65 %) males. The average age in the sample was: (males: mean 45.1 SD = 7.1; females: mean 45.3 SD = 65.9). The majority of participants self-identified as African-American 203 (76 %), followed by Hispanic 43 (16 %) and Caucasian 21 (8 %). A total of 190 (69 %) had completed high school. At baseline, participants provided recent (within one month from intake) lab tests of CD4 count and viral load. Lab reports showed viral load as undetectable for 128 (48 %) of the sample and an average CD4 count of 440 cells/mm^3^ (SD = 287). The overwhelming majority of participants, 219 (81 %) reported current use of antiretroviral medications, including Combivir, Emtriva, Epivir, Epzicom, Retrovir, Trizivir, Truvada, Videxec, Viread, Zerit, Ziagen, Crixivan, Invirase, Kaletra, Lexia, Novir, Prezista, Reyataz, Viracept, Intelence, Rescriptor, Sustiva and Viramune. Selection bias was not observed when participants’ characteristics in the main study were compared to those in the present study. Results suggest that participants were similar in age, education, sex, ethnicity and HIV clinical characteristics as shown in Table 2.

**Table 2:** Demographic and clinical characteristics of main study and current study participants

	Main study	Current study	P values
	n = 112	N = 267	
Age, mean (SD)	44.1 (7.7)	45.1 (7.1)	0.66
Sex, no (%)			0.72
Male	67 (60)	173 (65)	
Female	45 (40)	94 (34)	
Education no (%)			0.24
8th grade or less	13 (12)	19 (7)	
High school diploma	73 (65)	190 (69)	
Some college	26 (23)	57 (24)	
Race/ethnicity no (%)			0.26
Caucasian	17 (15)	21 (8)	
African–American	80 (72)	203 (76)	
Hispanic	15 (13)	43 (16)	
Alcohol use, mean (SD)			
Number of standard drinks (past 90 days)	100 (50.1)	190 (100.1)	0.10
Lifetime	22 (10.5)	23.8 (10.9)	0.24
AUDIT score	14 (7.5)	16 (8.0)	0.09
Other drugs, mean (SD)			
Number of times cocaine use (past 90 days)	23.5 (16.8)	33.5 (19.8)	0.25
Number of times marijuana use (past 90 days)	19.3 (12.5)	25.6 (20.9)	0.63
HIV characteristics, mean (SD)			
CD4 count	412.9 (318.4)	441.4 (286.9)	0.73
Viral load no (%)			0.16
Undetectable	45 (40)	128 (48)	
50–10,000	39 (35)	80 (30)	
10,001–30,000	8 (7)	29 (11)	
30,000 or more	20 (18)	29 (11)	
Taking ART	76 (68)	216 (81)	0.84
Cognitive measures, mean (SD)			
Executive skills *T*-*scores*	50.1 (9.0)	45.2 (10.9)	0.93
Memory skills (learning) *T*-*scores*	45.9 (10.1)	48.2 (9.1)	0.18
Memory skills (recall) *T*-*scores*	48.1 (9.8)	40.0 (10.5)	0.11
Cognitive flexibility *T*-*scores*	40.4 (10.4)	45.7 (10.8)	0.09
Visual memory *T*-*scores*	47.9 (11.9)	43.1 (13.8)	0.09

### Alcohol and other drugs of abuse

The TLFB determined alcohol and other drugs use. Questions included a total number of standard drinks consumed in the last 90 days, the total number of heavy drinking days (<5 standard drinks) in the last 90 days, and lifetime alcohol use. A standard drink is defined as 12 oz of beer, 5 oz of wine, 1.5 oz of liquor all of which contain approximately 13.6 g of absolute alcohol [46]. Results showed a mean AUDIT score of 16, which is categorized as a harmful drinking level. In addition, a total of 101 (38 %) of the participants scored >20 which is indicative of possible alcohol dependence. Lifetime alcohol use averaged 23.8 years for this sample. Additional detailed information on other substance use was also assessed. The main drugs used, besides alcohol, were cocaine and marijuana, with an average use in the last 90 days of 33 and 25 times, respectively.

The Frascati criteria were used to measure asymptomatic neurocognitive impairment, (1 standard deviation below the mean in at least 2 cognitive domains). Results for the neurocognitive measures were below average (*T*-*score*: mean 50; SD = 10). The cognitive domains with the lowest average scores were cognitive flexibility (mean 45.7; SD = 10.8) and executive function, (mean 45.2; SD = 10.9).

### DRD2 polymorphism and cognitive flexibility

Results of the analyses are presented in Tables 3 and 4. All SNPs were in Hardy–Weinberg equilibrium. The SNP rs6277 of *DRD2* gene showed an overall association with impaired cognitive flexibility (odds ratio = 1.6, 95 % CI 1.2–2.6; p = 0.004) and with executive function (odds ratio = 3.3, 95 % CI 2.0–5.7; p = 0.001). The association between SNP rs1800497 and cognitive flexibility was non-significant. Results were stratified by sex and race for cognitive flexibility and executive function. Testing showed an increased risk for executive function impairment in African Americans (odds ratio = 3.1, 95 % CI 2.3–3.5; p = 0.001), and an even greater risk for males (odds ratio = 3.5, 95 % CI 1.5–5.5; p = 0.008). There was, a significant gender interaction for cognitive flexibility (p_interaction_ = 0.013 for sex), but not for executive function (p_interaction_ = 0.35 for sex). At total of 40 (16 %) of participants carried SNPs rs6277 and rs1800497. Interaction with alcohol was not significant (p = 0.32) and no significant gene–gene interactions for *DRD4* and *DRD2* were found (results not shown).

**Table 3:** *DRD2* and *ANKK1* associations with cognitive domains

Chr.	Position	Gene	Variant	Minor Allele	A/A	A/B	B/B	MAF	Domain	OR_allele_^a^ (95 % CI)	P value
11	11:113412737	DRD2	rs6277	T	80	118	60	0.23	Cognitive flexibility	1.6 (1.2–2.6)	0.004
11	11:113412737	DRD2	rs6277	T	80	118	60	0.23	Executive function	3.3 (2.0–5.7)	0.001
11	11:113400106	ANKK1	rs1800497	T	102	117	40	0.16	Cognitive flexibility	1.1 (0.7–1.8)	0.71

ORs adjusted for self-reported ethnicity/race, alcohol use severity, viral load, CD4 count, cannabis and cocaine use

*MAF* minor allele frequency

^a^OR per allele (OR_allele_) for the additive model

**Table 4:** *DRD2* associations with cognitive flexibility and executive function in gender, race/ethnicity groups and alcohol use (ORs and 95 % CIs)

	Females	Males	Hispanics	African American	Alcohol use
DRD2 rs6277 (executive function)	1.3	3.5 (1.5–5.5) p = 0.008	2.6	3.1 (2.3–3.5) p = 0.01	2.6
	p_interaction_ = 0.35		p_interaction_ = 0.05		
*DRD2* rs6277 (cognitive flexibility)	0.9	1.8 (1.2–2.9) p = 0.01	1.9	1.5	1.6 (1.4–2.4) p = 0.03
	p_interaction_ = 0.013		p_interaction_ = 0.72		p_interaction_ = 0.32

### DRD4 48 bp VNTR polymorphism and executive function

The allele frequencies for *DRD4* 48 bp VNTR were similar to those observed in African populations in other studies [47, 48]. In this study, the most frequently detected alleles of the 48 bp VNTR of the D4 receptor were for *DRD4*-allele 4 (353/484, 72.9 %), and *DRD4*-allele 7 (66/484, 13.7 %). To a lesser degree *DRD4*-allele 2 (38/484, 7.8 %), *DRD4*-allele 3 (7/484, 1.5 %), *DRD4*-allele 5 (11/484, 2.3 %), and *DRD4*-allele 6 (9/484, 1.8 %) were also present. The nine and ten repeat alleles were not detected in this study population. The genotype distribution of the 242 participants is shown in Table 5. One hundred and eighty-six participants were grouped into the 7-absent allele group (<7 repeats), and 56 were grouped into the 7-present allele group (≥7 repeats). When comparing allele groups, the 7-allele present and 7-allele absent groups did not differ in sex, race/ethnicity, alcohol use or CD4 count. The 7-absent allele group mean score was associated with a higher rate of error in the Short Category Test measuring executive function than the 7-present group (mean 0.17, 95 % CI 1.17–1.29; p = 0.008). In addition, a multiple linear regression with executive function as the dependent variable and age, sex, alcohol use, genotype group and race/ethnicity as the independent variables showed that *DRD4* 7-absent allele and age had a significant effect on executive function. Whereas, sex, alcohol use and race/ethnicity did not show a significant effect (data not shown).

**Table 5:** D4 Receptor 48 bp repeat genotype group classification

D4 receptor 48 bp repeat genotype	N	%	Genotype group
2/2	8	3.3	1
2/3	2	0.8	1
2/4	20	8.4	1
3/4	2	0.8	1
3/6	3	1.0	1
4/4	140	57.8	1
4/5	5	2.0	1
4/6	6	2.6	1
4/7	40	16.7	2
5/7	6	2.4	2
7/7	10	4.2	2

*Group 1* 7-absent group: <7-fold repeat of the 48 bp repeat of D4 receptor, *Group 2* 7-present group: ≥ 7-fold repeat of the 48 bp repeat of D4 receptor

## Discussion

This study provides evidence that suggests genetically determined differences in *DRD2* gene polymorphism (rs6277) and *DRD4* gene (48 VNTR) are associated with impaired executive function and cognitive flexibility. However, no associations were found with SNP rs1800497. It is well-recognized that genes are likely to affect more than one cognitive function, and variations in cognitive functions are likely to be influenced by more than one gene [49]. Similarly, this study showed that the DRD2, SNP rs6277 is associated with impairment in two cognitive domains: executive function and cognitive flexibility. Conversely, executive function is influenced by *DRD2* and *DRD4* genetic polymorphisms. Although recent publications stress the need to consider gene–gene interactions, our results showed no such interactions [49].

We showed that SNP rs6277 C/C-carriers were less efficient in task switching as it took them more time to complete the Color Trails Test than T/T and C/T carriers. Similarly, the total number of errors in the Short Category Test was higher for C/C-carriers representing poorer executive function. Our results are in line with others that reported an association between C-homozygotes and poorer executive functioning and memory [50, 51], and lower cognitive ability in C/C-carriers measured in five different cognitive domains [52]. However, other studies showed that T-homozygotes are associated with dysfunctional impulsivity [53] and that carriers of at least one T-allele showed a significantly poorer performance in the identification of T1 in the Attentional Blink phenomenon [54]. These differences may be related to SNP rs6277 in the *DRD2* gene that changes the receptor’s affinity and regulates the *DRD2* availability, but its effect differs depending on the brain region under investigation [55, 56].

The clinical implications for the role for the *DRD2* SNP rs6277 has been associated to learning [57], reward sensitivity [58], substance abuse [59–61], nicotine modulation of working memory [62], pharmacological interventions [63, 64] as well as in schizophrenia [24, 65, 66]. Altogether, this evidence suggest a reasonably and significant role for the SNP rs6277 in psychiatric disorders. Thus, *DRD2* SNP rs6277 may also play a role in executive function and cognitive flexibility in patients with HAND.

The *DRD4* 48 bp VNTR polymorphism has been previously linked to Attention-deficit/hyperactivity disorder (ADHD) phenotypes [10, 67–70]. In particular, the specific allele (7-repeat) of the 48pb VNTR polymorphism in the coding region of DRD4 may be a risk factor in the development of ADHD [10]. ADHD is known to alter prefrontal cognitive functions that are often related to dopaminergic dysfunction [71]. Thus, following previous studies on ADHD, this study sought to assess whether cognitive functions (cognitive flexibility and executive function) were associated with the *DRD4* 48 bp VNTR polymorphism in HIV-infected adults. Results showed that the 7-absent allele group was significantly associated with executive dysfunction. The effect of the *DRD4* VNTR on executive function reported herein is comparable with a familial study that reported a significant association between the 7-absent allele group and lower scores in working memory and executive function [67]. Similarly, several studies on *DRD4* VNTR showed that *DRD4* 7-absent allele group was associated with worse cognitive functioning than the *DRD4* 7-present allele group [69, 72]. However, the results of this study are in conflict with the findings of other similar studies. One study found poorer inhibitory performance in the 7-present allele group versus the 7-absent allele group [68]. Another, found that 7-present allele group performed better that the 7-absent allele group on verbal memory, but for visuo-constructive ability and set shifting the 7-absent allele group performed better than the 7-present allele group [73]. This poses important questions with respect to the relationship between genetic risk and neurocognitive performance. There are several potential explanations for these conflicting results. Including higher and lower than average levels of synaptic dopamine may lead to neurocognitive impairment [74]. This is a particularly interesting since the 7-present allele is associated with reduced receptor functioning [73]. The combinations of certain risk genotypes rather than one single risk genotype may lead to the presence of cognitive dysfunction [75]. These relationships have not been fully tested and require further research, especially since cognitive endophenotypes are important for HIV-associated neurocognitive impairments.

It is important to note limitations that restricted conclusions in certain areas. First, due to the exploratory nature of the study, multiple statistical comparisons were made. Because of the low power of the study to detect smaller effect sizes, some important associations may not have emerged as statistically significant. These results should be viewed with caution and should be replicated before a definitive conclusion can be drawn. Second, due to the vast number of HIV antiretroviral drugs used by study participants, we did not adjust for HIV medication type. Since certain HIV antiretroviral drugs may also affect cognition, this may potentially confound the results. Third, two main approaches are used to approximate individual ancestry in association studies, self-reported race and ancestry informative markers. We did not use ancestry informative markers due to DNA requirements. Instead, we used self-reported ancestry that may capture common environmental influences as well as ancestral background. However, self-identified racial categories may not always consistently predict ancestral population clusters. Finally, since this was a cross-sectional study stemming from a behavioral intervention trial of HIV-infected subjects, we did not have a healthy control group. Although we adjusted for alcohol and drug use, the results may not adequately explain whether impairments in cognitive flexibility and executive function were correlated with the presence of SNP rs6277 and VNTR 7-absent allele or mediated by HIV and alcohol/drug use. Nonetheless, these results may serve as an initial point for future research in cognitive phenotypes for HAND in adults. Molecular genetics, as applied in the present study, offers further analytic insight beyond behavioral assessment and neuroimaging, and may present a reasonable instrument for the differentiation of executive control processes.

This study may pave the way for future research integrating the examination of genetic factors in behavioral prevention interventions with HIV-infected populations. Studies that incorporate genetic factors in combination with neurocognitive testing would benefit from also including the effects of genetic factors on cognitive functioning in healthy individuals since gene-by-disorder interactions might be expected. Furthermore, it would be beneficial to investigate haplotypes rather than genotypes in studies on cognitive performance in HAND. Since most of the polymorphisms have a small relative effect on cognition, to detect an effect, a larger sample is optimal. In addition to the genes analyzed in this study, other genes related to cognitive function should be included.

In summary, the present study provides evidence that genetically determined differences in genes *DRD2* SNP and *DRD4* 48 bp VNTR may contribute to deficits in executive function and cognitive flexibility for patients with HAND. Additionally, rs6277 showed an association with impairment in two cognitive domains (executive function and cognitive flexibility) while executive function seemed to be influenced by *DRD2* and *DRD4* genetic polymorphisms. Finally, *DRD4* 48 bp VNTR (7-allele absent group) was associated with executive dysfunction, which is in line with the recent suggestion that either higher or lower levels of synaptic dopamine may lead to neurocognitive impairment.
